# Implementing a court diversion and liaison scheme in a remand prison by systematic screening of new receptions: a 6 year participatory action research study of 20,084 consecutive male remands

**DOI:** 10.1186/1752-4458-7-18

**Published:** 2013-06-25

**Authors:** Clare McInerney, Mary Davoren, Grainne Flynn, Diane Mullins, Mary Fitzpatrick, Martin Caddow, Fintan Caddow, Sean Quigley, Fergal Black, Harry G Kennedy, Conor O’Neill

**Affiliations:** 1 National Forensic Mental Health Service, Central Mental Hospital, Dundrum 14, Dublin, Ireland; 2 Department of Psychiatry, Trinity College, Dublin, Ireland; 3 Irish Prison Service, Dublin, Ireland

**Keywords:** Psychiatric illness, Prison psychiatry, Screening, Court liaison, Court diversion

## Abstract

**Background:**

A mental health needs assessment in the Irish prison population confirmed findings from other jurisdictions showing high prevalence of severe mental illness, including psychosis amongst those newly committed. We implemented a participatory action research approach in order to provide an integrated mental health prison in-reach and court liaison service for this population.

**Results:**

Following extensive consultation, a two stage screening process was developed which was supplemented by an inter-agency referral management system. During the six years 2006–2011, all 20,084 new remands to the main remand prison serving 58% of the national population were screened. Following the first stage screen, 3,195 received a comprehensive psychiatric assessment. Of these 561 (2.8%) had symptoms of psychosis – corresponding to the prior research finding – and 572 were diverted from the criminal justice system to mental health services (89 to a secure forensic hospital, 164 to community mental health hospitals and 319 to other community mental health services).

**Conclusions:**

We have shown that it is possible to match research findings in clinical practice by systematic screening, to sustain this over a long period and to achieve consistent levels of diversion from the criminal justice system to appropriate mental health services. The sustained and consistent performance of the model used is likely to reflect the use of participatory action research both to find the most effective model and to achieve wide ownership and cooperation with the model of care.

## Background

Diversion has been defined as a policy of transferring the mentally ill away from the criminal justice system and into psychiatric care [1,2]. Some writers have limited the definition of diversion to the provision of inpatient admissions, reserving the term “liaison” for non-inpatient community treatment arrangements [2,3]. While there are many descriptions of effective police-station and court-based diversion services for local areas, reviews of diversion services have highlighted inequalities between local areas, and the need for standardisation of approach to enable equal access over larger geographical areas and population aggregates [2-6].

Policy and expert guidelines exist to advise on the organization of mental health services in prisons [4-6]. The UN Declaration of Rights of the Mentally Ill [7] emphasises that persons with major mental illness should have equal rights with their non-mentally ill counterparts, including equal rights to bail and liberty. The UN Principles for the Treatment of Prisoners [8] states that prisoners should have access to the health services available in the country “without discrimination on the grounds of their legal situation”. Similarly, the Council of Europe Committee for the Prevention of Torture (CPT) has emphasized the need for equivalence of care for prisoners with persons in the community [9].

A 2012 systematic review and meta-analysis of 33,588 prisoners from 24 countries [4] found a pooled prevalence rate of psychosis of 3.6% (95% CI 3.1-4.2) in male prisoners and 3.9% (95% CI 2.7-5.0) in female prisoners. The authors observed that the prevalence of psychosis appeared to be stable over time.

A 2002 cross-sectional study of Irish male remand prisoners found a six month prevalence of psychosis of 7.6% [10]. A committal survey of newly-remanded male prisoners in the same prison in Ireland involving a large sample found that 3.8% had active symptoms of psychotic illness [11]. In the ten years since these Irish studies, the mean daily prison population in Ireland increased by 39% from 3,165 in 2002 to 4,390 in 2011 [12,13]. Total committals increased by 46% from 11,860 in 2002 to 17,318 in 2011 [12,13].

Persons with major mental illness coming in contact with the criminal justice system should be identified as early as possible, ideally at the point of arrest or at first court appearance. Reliance on identification of persons with major mental illness and consequent referral by criminal justice workers has not been shown to be effective [14]. Screening instruments have been validated in prison settings [15,16]. The utility of such instruments may be limited in everyday clinical practice. Persons with acute paranoid symptoms may not respond to screening questions. There is a limited research base regarding the longitudinal effectiveness of such processes in clinical practice for large population aggregates over extended periods of time. Limitations in data reporting of diversion schemes have been described [2,3,6,17].

The role of participatory action research in implementing change in mental health service delivery in prisons has been described recently [18]. We set out in 2006 to apply the same methodology to the development of a prison in-reach and court liaison service in Ireland’s busiest prison for men remanded into custody prior to trial.

### Rationale and objectives

This project was driven by two needs. Firstly there was a need to implement a standardized system for identification of persons with major mental illness remanded to Ireland’s main remand prison, with audit procedures for comparison with predicted rates based on epidemiological evidence [10,11]. Secondly there was a need to implement systems to enable diversion to appropriate health care settings of mentally ill persons identified.

## Methods

### Case study

This study describes the effect of introducing a dedicated prison inreach service to Ireland's main remand prison. The role of such a service is to identify persons with major mental illness as rapidly as possible and to broker “joined-up” care for patients, liaising between the patient, community psychiatric services, the judiciary and correctional staff.

### Drivers of change

In response to criticisms by the Council of Europe Committee for the Prevention of Torture [19] there was new investment in staff and resources for the national forensic mental health service. These resources enabled needs assessment surveys to be carried out in the Irish prison population examining both the cross-sectional and point prevalence of severe mental illness and substance misuse [10,20] and the dynamic rates of reception of cases of severe mental illness in remand and sentenced prisons [11].

In 2005 a national policy for the modernisation of mental health services, *A Vision for Change*[21] provided principles for the care and treatment of those mentally ill persons in contact with the criminal justice system incorporating existing international principles such as the need to provide care for the mentally ill offender in their community mental health service in so far as possible and the need to treat the mentally ill in the least restrictive placement compatible with their needs and safety. A *Vision for Change* further recommended the development of court diversion services.

The lack of a modern system for diverting the mentally ill from the prisons to mental health services prompted the next stage in service development described in this case study.

### Setting

In 1999 a new prison opened in Dublin which centralised the reception of remand (pre-trial) prisoners for the majority of the population of the Republic of Ireland.

During the period under study (2006–2011), the prison received remands from courts serving a geographical “footprint” covering the majority of the national population. The prison received remands from courts covering 57.0% of the population in 2006 (2,415,123/4,239,848) [22] and 57.5% of the population in 2011 (2,638,480/4,588,252) [23]. During the six years of this study the prison received 58.5% (20,084/34,323) of all remands nationally (Table 1).

**Table 1:** Committals on remand, trial, deportation and extradition 2006–2011 to Cloverhill and to all prisons nationally

	**Persons**		**Committals**	
**Year**	**All institutions**	**Cloverhill**	**All institutions**	**Cloverhill**
2006	5,165	3,233	6,515	4,107
2007	5,024	2,882	6,219	3,562
2008	4,371	2,674	6,096	3,635
2009	3,580	2,103	5,199	2,919
2010	3,480	2,038	5,318	3,121
2011	4,236	2,369	4,976	2,740
**Total**	**25,856**	**15,299**	**34,323**	**20,084**

Source: Irish Prison Service.

Prior to 2006, prison psychiatric inreach in Ireland involved the provision of sessional clinics by individual medical staff from the national forensic mental health service, whose primary roles lay elsewhere. Severely mentally ill prisoners when identified, were placed in a landing above the medical area. Also placed on the same landing were vulnerable prisoners and those with significant physical illnesses. This model had limited capacity to coordinate and follow through on treatment plans. Rapid throughput in remand prisons meant this model was able to provide only limited continuity of care, leading to frequent loss to follow up within the prison and criminal justice system and difficulty ensuring that prisoners received timely treatment in appropriate settings. Identification of mental illness was based almost entirely on referrals from prison nursing staff following unstructured health screening on reception.

The mental health service for the new remand prison was designed to identify all those with psychosis on reception and to divert those with severe mental illness requiring treatment outside a prison setting at the earliest opportunity.

### Legal context

Many countries have introduced specific and comprehensive mental health criminal law legislation to provide for this. Ireland, at the time of writing, has very limited legislative provision to protect the rights of the mentally ill coming in contact with the criminal justice system.

Legal provisions for psychiatric care in Ireland are set out in the Mental Health Act 2001 [24] which commenced in November 2006 for civil committals, and in the Criminal Law (Insanity) Act 2006 [25], which commenced in June 2006. For mentally ill people charged with offences, Section 12 of the Mental Health Act 2001 allows police to directly divert from the criminal justice system to mental health settings by initiating an application for assessment under the (civil) Mental Health Act. While Ireland does not yet have specific legislation to provide for court diversion to community settings or involuntary community treatment, the process can take place within existing mental health law, along with bail and probation legislation.

The Criminal Law (Insanity) Act 2006 (section 15) provides for transfer of prisoners to the Central Mental Hospital, voluntarily or involuntarily, where the person suffers from a mental disorder as certified by two medical practitioners. Section 15 does not provide for transfer of persons with mental illness to psychiatric facilities other than the Central Mental Hospital. Judges may order transfer of defendants found to have a mental disorder and to be unfit to be tried to a ''designated centre'' under section 4 of the Criminal Law (Insanity) Act 2006. The Central Mental Hospital, Ireland's only forensic psychiatric hospital, is the only centre so designated and represents an inappropriately restrictive option (high and medium security) for many patients appearing before the courts, particularly those charged with minor offences. The Criminal Law (Insanity) Act 2006 at Section 4(7) anticipates this in that the court ''may defer consideration of the question (of fitness) until any time before the opening of the case for the defence” [25].

### Stakeholder consultation

Service users in Irish prisons had been extensively surveyed for mental health needs prior to the implementation of the service [10,11,20,26]. Carers’ groups pointed out the need to enable access to services at the earliest possible juncture.

Members of An Garda Siochana (the Irish police force) in 2006 described difficulties in obtaining medical assessments in police stations and the long delays when mentally ill persons were brought to emergency departments of acute hospitals or to psychiatric hospitals.

At a national meeting in 2006 to guide development of the service, District Court judges reported frustration at the frequent appearance of visibly disturbed individuals charged with minor crimes, for whom there appeared to be no coherent system of rapid referral to psychiatric services for assessment.

Prison governors were dissatisfied that, no matter how minor the charge, the Criminal Law (Insanity) Act only permitted the transfer of prisoners in need of psychiatric hospital care to the Central Mental Hospital, the national high and medium secure hospital, where there were long waiting lists.

General adult mental health services expressed dissatisfaction regarding the manner in which patients came to treatment from the courts; in particular problems were experienced with inappropriate referrals of patients (particularly persons with addiction problems only) who presented to hospital with limited notice and inadequate background information.

### Service innovation

The Prison Inreach and Court Liaison Service (PICLS) was initiated by the National Forensic Mental Health Service (part of the Department of Health’s Health Service Executive, the state’s public health serviced) to address these shortcomings in 2006, with a full team in place by 2007.

### Options considered

Ideally such a service would identify and divert patients at the point of arrest or in police stations before they entered custody, thus reducing the number of mentally ill minor offenders entering prison. Ireland has a predominantly rural population with many local police stations. Similarly a daily, or even weekly, presence in all District Courts was impossible to provide for geographical and resource reasons and because any one police station or any one court would have only a few cases presenting sporadically.

While a referral system to one or more dedicated mental health courts was considered, any such referral system would be likely to involve a period of remand in custody from the referring courts and significant delays from referral to diversion have been previously reported [27].

For reasons of practicality and equitability, the decision was taken to base the service in Cloverhill Prison, Ireland’s main remand prison, which received the majority of remands nationally during the period studied, as described above.

### The prison in-reach and court liaison model

A multi-disciplinary mental health team, consisting of three medical staff (one consultant forensic psychiatrist and two psychiatric trainees, post-membership psychiatrists equivalent to north American ‘fellows’) and three nursing staff (all experienced clinicians at Masters level) was established on site at the prison to provide a full-time Monday to Friday service. There was also one team administrator, making 5.4 whole time equivalents in all. The service was fully established from 2007. The service aims to identify mentally ill prisoners as rapidly as possible, and put in place practical solutions for accessing appropriate mental healthcare. The service operates a “liaison” model, with assertive efforts to link patients to their local psychiatric service when this is feasible and safe.

The aim of the Prison Inreach and Court Liaison Service is to assist patients, the criminal justice system and local psychiatric services by identifying mentally ill persons when remanded to prison as rapidly as possible, and to put in place practical solutions for accessing appropriate mental healthcare. The service aims to identify those with a primary diagnosis of psychotic illness, including those with co-morbid substance misuse problems. Separate parallel services for persons with ''pure'' substance misuse difficulties are provided by the visiting prison addiction services and the drug courts.

In practice the process involves a number of steps as follows:

### Prison health screening on committal

All prisoners receive a standard committal interview on reception (typically within two hours of reception) carried out by Irish Prison Service nursing staff, based on review of validated screening instruments used in other jurisdictions [15,16] and modified for the Irish prison population. In order to achieve maximum detection of major mental illness, a further daily screening process by mental health staff was introduced. Screening consists of selecting for interview all committals who on reception disclose to the interviewing nurse a history of previous psychiatric contact or prescription of psychiatric medication, a history of deliberate self-harm, or who exhibit unusual behaviour which may require special placement within the prison. Persons charged with homicide are assessed, as are individuals with a known past history of treatment by prison psychiatric in-reach services. This modified screening instrument is treated as a structured professional judgement instrument: those who deny all symptoms and history but who are evidently disturbed will be referred for assessment.

The provision of a dedicated team with consistent staffing over time assists in identifying persons previously committed by systematic review of computerized case notes. In addition to this screening process, referrals are accepted from a wide range of sources including prison general practitioners, nursing and correctional staff, judiciary, legal representatives, police and probation staff, family and friends.

### Allocation to a high observation area

Those identified as severely mentally ill or otherwise in need of high support from prison nursing and medical staff are placed in a landing for vulnerable prisoners. The high observation area is half of one wing, consisting of 15 beds in single and double cells. There are also safety observation cells used in exceptional circumstances for those posing a serious and immediate risk to themselves or others in the context of major mental illness. Remanded prisoners are placed on the high observation area following reception at the discretion of the prison governor, in consultation with prison medical and nursing staff. The general practitioners and general nurses employed by the prison service do rounds there every day. The staff of the psychiatric in-reach team (PICLS) attend there five days a week. This allows higher levels of observation by medical and nursing staff than would be possible on ordinary prison locations and higher levels of clinical support. The discipline staff (prison officers) receive special training concerning mental health when allocated to work there. This training emphasizes the caring role as part of good order and discipline and there is particular vigilance regarding bullying and intimidation.

### Assessment, case management and interdisciplinary working

Patients are assessed initially and on subsequent reviews by pairs of key working medical and nursing clinicians. This aids in continuity of care. One clinician interviews the patient while the other records the computerized notes which are reviewed before saving. Given that time windows for patient assessments in prison are often limited to non-“lockdown” periods, this means more people can be seen in the time available. For similar reasons, patients are interviewed in designated interview rooms on the wing of the prison in which the patient is situated. Team workers have found that in other large prisons, waiting for a patient to be brought from wings or recreation areas to a single centralized waiting area can be time-consuming.

### Multidisciplinary and interdisciplinary team meetings

A weekly “dry round” is conducted to discuss and develop case management plans for all patients. Structured case management plans are updated and entered into the computerized patient record after each meeting.

The “dry round” is followed by an interdisciplinary meeting of PICLS staff and other prison workers, including prison nursing staff, (prison nursing staff and general practitioners are employed directly by the Irish Prison Service rather than by the state’s Health Service Executive, the public health service), the prison governor, probation services, the prison chaplain and addiction counselling services to ensure continuity of care. The next court date is reviewed and contingency plans are made and implemented for management after release on bail or other non-custodial disposal together with management plans in the event of the person’s remaining in prison.

Newly-remanded prisoners typically have their first court date within seven days of initial committal. Weekly e-mails are sent by prison management to the PICLS team with a copy of the list of persons applying for high court bail to ensure contingency plans are in place for persons who may be released in advance of their next scheduled court date.

### Triage options

When a person detained in an Irish prison requires psychiatric treatment, several triage options exist. Treatment in the patient’s catchment area service often represents the best option for individuals with severe mental illness charged with a minor offence, and deemed to pose low risk to others. In such instances the court may prefer to impose bail or other non-custodial disposal rather than drop charges. This allows for conditions to be put in place to promote compliance with psychiatric treatment. Persons receiving bail, whether or not suffering from a mental illness, are generally expected to comply with specific conditions, providing for a balance between rights and responsibilities.

Transfer to the Central Mental Hospital, Ireland’s only secure forensic hospital is also an option. The Central Mental Hospital, like the PICLS team, is part of the National Forensic Mental Health Service, an integral part of the Health Service Executive, the state’s health service. The Central Mental Hospital serves the entire country and provides treatment in conditions of high, medium and low security. Transfer to the Central Mental Hospital should be reserved for persons with severe illness who are thought to pose a high risk to others, and would not be suitable for management in a general adult psychiatric inpatient setting [28]. In-reach treatment in the prison setting is often suitable for persons with less severe mental illness [29].

### Liaison: communication, networking and planning

Liaison with all relevant stakeholders begins at the point of identification as in need of mental health assessment. This includes communication with the individual’s relatives, general practitioner, the arresting police officer and the person’s community psychiatric service and other relevant stake holders. This requires the consent of the person concerned. Where the person is assessed as lacking the mental capacity to give or withhold consent, the clinicians are obliged to act in the person’s best interests. This may involve alerting the person’s legal representative to their mental health needs.

Information gathering at this stage pays particular attention to the individual’s past psychiatric history and history of previous violence in order to assess the potential level of risk posed. If it is anticipated that the individual may receive bail at the next court appearance, early contact is made with the relevant community psychiatric team in order to arrange appropriate treatment.

### Court appearance, court reports and transfer arrangements

Where a defendant before the court with severe mental illness has urgent treatment needs, and a likely eligibility for bail, it is usual practice to provide a psychiatric report to the court and a comprehensive referral letter to the receiving psychiatric team for the patient's catchment area. Such reports are prepared voluntarily and on request with the consent of the person concerned. As before, where the person is assessed as lacking capacity to give or withhold consent, the clinicians may be obliged to act in the person’s best interests, informing the court of the person’s mental health needs as a minimum. In addition to a detailed history supplemented by collateral information, reports conclude with advice regarding diagnosis, current mental state, fitness to be tried and treatment arrangements that are in place (rather than ''recommendations'') in the event of custodial and non-custodial disposal.

**Figure 1:**
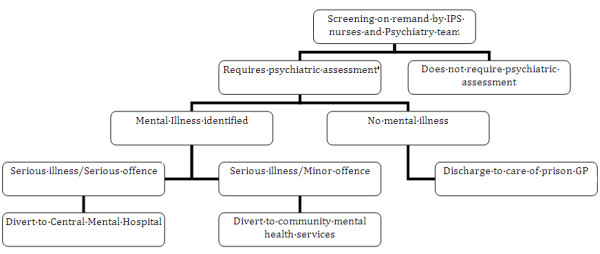
Prison inreach and court liaison service model flowchart.

In the case of a seriously ill person requiring involuntary psychiatric treatment, Mental Health Act documentation for certification is prepared in advance of the court appearance. Where admission to a local psychiatric hospital is recommended, defendants are accompanied to court by a member of the Prison Inreach and Court Liaison nursing staff to provide oral evidence if required and assist in transfer to hospital from court, where necessary. The process is summarized in Table 2 and Figure 1.

**Table 2:** The Psychiatric prison inreach and court liaison model: summary of PICLS process

	
**1. Screening: identification of prisoners with severe mental illness**	**Daily screening of prison committals**
	● Previous psychiatric contact
	● Psychiatric medication
	● History of self-harm
	● Homeless
	● Observed unusual behaviour
	● Charged with homicide or arson
	● Referrals from courts, prison staff community
	● services and other sources
**2. Triage: Identification of Appropriate Treatment Options**	**Major offence: major mental illness**
	● Transfer to Central Mental Hospital
	**Minor offence: major mental illness**
	● Inpatient or outpatient community treatment in event of bail
	or other non-custodial disposal
	**Major offence: minor mental illness**
	● Follow-up in prison
	**Minor offence: minor mental illness**
	● Prison follow-up
**3. Collateral History & Liaison: Continuity of care**	● Relatives
	● Community psychiatric services
	● Garda Siochana (Police)
	● Probation and welfare services
	● Solicitor
	● Homeless agencies where relevant
	● Other agencies as required
	● Daily liaison with prison nursing staff
	● Weekly case management meetings of all patients actively managed by service.
	● Weekly case review meetings with prison medical, nursing and other staff.
**4. Court appearance**	● Court report
	● Staff present in court where needed for communication and assistance in transfer to hospital
	● Bail conditions sought
**5. Treatment**	● Diversion to appropriate treatment location

### Study method

All new remands to Cloverhill Prison during the six years from January 1st 2006 to December 31^st^ 2011 were screened as described above. Cases were identified prospectively and recorded by PICLS staff at the time of clinical assessment using pro-forma sheets/computerized records. Anonymised information was analysed using SPSS 19. The data recorded is that routinely collected for the annual report of the service.

## Results

### Case mix

There were 20,084 new committals (defined as committals on remand, trial, deportation and extradition) of 15,299 individuals to Cloverhill Prison during the period January 1^st^ 2006 to 31^st^ December 2011 (Table 1). This constituted 58.5% (20,084/34,323) of all remands nationally during this 6 year time period. Overall 15.9% (3,195/20,084) of new remands, made up of 2,368 individuals were identified by the screening and referral process and received comprehensive assessment during this six year period.

### Demographic and social variables

Table 3 shows that all remands were male. Mean age at first assessment was 31.8 years (SD 10.18: N=3,195). Of remands assessed, 84.2% (2,690/3,195) were Irish, 7.9% (251/3,195) from other EU countries and 7.9% (254/3,195) from countries outside the EU. There were 23.4% homeless (748/3,195) with 73.9% (2,362/3,195) not homeless and 2.7% (85/3,195) unknown.

**Table 3:** Case mix: historical and clinical variables for cases (N=3,195)

**Gender**	**Male sex**	**3195**	**(100%)**
**Age (Mean/SD)**	31.8 years	(SD 10.18)	
**Nationality**	Irish	2,690/3,195	84.2%
	other EU	251/3,195	7.9%
	non- EU	254/3,195	7.9%
**Homeless**	Homeless	748/3,195	23.4%
	Not homeless	2,362/3,195	73.9%
	Unknown	85/3,195	2.7%
**Substance misuse**	Any	2,773/3,195	86.8%
**Substance misuse**	Alcohol alone	501/3,195	15.7%
	Drugs alone	827/3,195	25.9%
	Alcohol and drugs	1,445/3,195	45.2%
	Neither	346/3,195	10.8%
	Unknown	76/3,195	2.4%
**Past primary diagnosis (lifetime)**	Any psychosis	705/3,195	22.1%
	Mood/anxiety	754/3,195	23.6%
	No major illness	1,555/3,195	48.6%
	Unknown/other	181/3,195	5.7%
**Lifetime psychotic symptoms (including drug-induced)**	Yes	949/3,195	29.7%
	No	2,211/3,195	69.2%
	Unknown	35/3,195	1.1%
**Current primary diagnosis**	Any psychosis	766/3,195	24.0%
	Mood/anxiety	480/3,195	15.0%
	Substance withdrawal	88/3,195	2.8%
	No major illness	1,818/3,195	56.9%
	Unknown/other	43/3,195	1.3%

Based on screening and subsequent assessment of the remands assessed, 705 (22.1%) had a previous primary diagnosis of any psychotic condition. A history of mood or anxiety disorders was elicited in 754 (23.6%), 77 (2.4%) had a previous primary diagnosis of personality disorder and 40 (1.3%) of learning disability in the absence of psychotic or mood/anxiety disorder. Overall, 1,555 (48.6%) had no major mental illness diagnosed and 64/3,195 (2.0%) had previous diagnosis recorded as “unknown/other”.

Based on assessment and collateral history, 949/3,195 (29.7%) of cases were found to have a lifetime history of psychotic symptoms while 2,211 (69.2%) had no recorded history of psychotic symptoms, with 35 (1.1%) recorded as “unknown”.

A history of substance misuse was elicited in 86.8% (2,773/3,195) based on screening and subsequent assessment. A history of alcohol abuse was present in 501/3,195 (15.7%) while 827/3,195 (25.9%) had a history of misuse of other substances and 1,445/3,195 (45.2%) a history of misuse of both alcohol and other substances. Only 346/3,195 (10.8%) had no recorded history of substance misuse, with 76/3,195 (2.4%) unknown.

### Clinical variables

Table 3 shows that using ICD-10 criteria 665/3,195 (20.8%) of cases assessed received a diagnosis of schizophreniform psychosis, 69 (2.2%) of affective psychosis and 32 (1.0%) of organic psychosis, 24% in all. The primary diagnosis was mood/anxiety disorder for 480/3,195 (15.0%) while 88/3,195 cases (2.8%) were diagnosed with substance withdrawal conditions. No major mental illness was diagnosed in 1,818/3,195 (56.9%) while. 43 (1.3%) received diagnoses recorded as “unknown/other”.

### Outcomes: Identification of persons with active psychotic symptoms

Table 4 shows that 561/20,084 (2.8%) of consecutive receptions over the six years were identified using the multistage screening and referral process as having psychotic symptoms (delusions, hallucinations or thought disorder) during their remand period at Cloverhill. This equates to 17.6% (561/3,195) of cases fully assessed following the reception screen, as exhibiting active psychotic symptoms while on remand. A total of 2,633/3,195 (82.4%) were recorded as not exhibiting such symptoms, with one unknown.

**Table 4:** Outcomes (N=20,084)

			
**Identification of psychosis (all: N=20,084)**	Psychotic symptoms identified during remand	561/20,084	(2.8%)
**Identification of psychosis (cases: N=3,195)**	Yes	561/3,195	17.6%
	No	2,633/3,195	82.4%
	Unknown	1	-
**Assessed based on Screening and referral**	Assessed	3,195/20,084	15.9%
	Did not require assessment	16,889/20,084	84.1%
**Psychiatric outcome**	Forensic admission	89/20,084	0.44%
	General Psychiatric admission	164/20,084	0.82%
	Other community settings	319/20,084	1.58%
	Discharged to prison GP/addiction service	1,700/20,084	8.46%
	Prison transfers	889/20,084	4.43%
	Remained under psychiatric care at 31.12.11	19/20,084	0.09%
	Did not require assessment	16,889/20,084	84.1%
	No recorded outcome	14/20,084	0.07%

### Outcomes: diversions and other psychiatric disposals

As at 31^st^ December 2011, 89/3,195 (2.8%) of remands assessed (0.44% of all committals) were admitted to the national forensic psychiatry unit over the six year period of observation. Table 4 shows that 164/3,195 (5.1% of those assessed, 0.82% of all committals) were admitted to local (general psychiatry) psychiatric units, of which 137/164 (84%) were admitted under the Mental Health Act, and 27/164 (16%) as voluntary patients. 319 (10.0% of those assessed, 1.58% of all committals) were diverted to other community treatment settings, including psychiatric outpatient departments, supported residences, residential rehabilitation facilities and nursing homes. Overall, 2.84% of all committals over the six year period were diverted from prison to psychiatric care, in hospital or the community.

Table 4 shows that at the end of the observation period 19/3,195 (0.6%) remained on remand at Cloverhill plus a further 3 who had been returned to the prison following admission and treatment at the national forensic psychiatry unit.

A total of 1,700/3,195 (53.2%) were discharged to the prison GP and/or addiction services, 889 (27.8%) were transferred to other prisons while 14/3,195 (0.5%) did not have a recorded outcome.

Table 5 shows that the proportion of committals found to have a psychosis was fairly constant year on year and fell within the confidence interval of the research-standard estimate given by Curtin et al. for current prevalence rate of any psychotic illness of 3.8% (95% confidence interval 2.2% to 6.6%) based on a survey in 2004 [11].

**Table 5:** Annual numbers found positive for active psychotic symptoms as a percentage of all committals compared to the proportion diverted from prison to mental health services

	**All committals**	**Active psychotic symptoms**		**Number diverted from prison to mental health services**	
**Year**	**Number**	**Number**	**% (95% CI)**	**Number**	**% (95% CI)**
2006	4,107	95	2.3(1.9-2.8)	60	1.5(1.1-1.9)
2007	3,562	102	2.9(2.4-3.5)	87	2.4(2.0-3.0)
2008	3,635	112	3.1(2.6-3.7)	118	3.3(2.8-4.0)
2009	2,919	70	2.4(1.9-3.0)	115	2.9(2.5-3.5)
2010	3,121	91	2.9(2.4-3.6)	110	3.5(2.9-4.2)
2011	2,740	91	3.2(2.6-3.9)	82	3.0(2.4-3.7)
Total	20,084	561	2.8(2.6-3.0)	572	2.8(2.6-3.1)

Table 5 also shows that the proportion of committals diverted to mental health services was fairly constant year on year, with a close correspondence between the two. Only in 2006, when the service was incomplete, did the number diverted fall significantly below the number with psychosis. The numbers identified and diverted each year fell within the confidence interval for the expected prevalence of psychosis given by Curtin et al. [11] except for the first year, 2006.

### Analysis of productivity

The team consisted of 5.4 whole time equivalents, though for much of the time fewer staff were in post. Even assuming full staffing, this equates to 17.3 diversions per whole time equivalent per annum, and 98.6 service users assessed and case managed per whole time equivalent per annum as a result of the screening and assessment service. There were many other clinical contacts with prisoners in ordinary prison locations (wings) and in the high observation unit as well as involvement in clinical liaison and administrative contacts with prison governors and primary care medical and nursing staff.

## Discussion

### Key results

The systematic screening of all newly received remand prisoners identified 2.8% (561/20,084) as having a current psychosis. This meant psychotic symptoms based on repeated interviews and collateral history. This compares with the finding of Curtin et al. [11] in the same prison in 2004 that 3.8% (95% confidence interval 2.2% to 6.6%, n=313 interviewed) had a current psychosis using a research diagnostic interview and prison medical records for a systematically ascertained sample of successive committals. We are satisfied therefore that the two stage screening process is capable of matching research standard methods.

**Figure 2:**
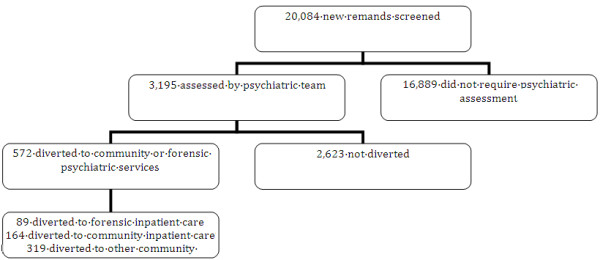
Outcomes of diversion process flowchart.

We succeeded in diverting from prison to mental health services a total of 572 (Figure 2) prisoners with severe mental illness over the six year period observed. These were delivered to varying levels of care (89 to a secure forensic hospital, 164 to community mental health hospitals, 319 to other community mental health services) demonstrating risk-need responsivity. We have elsewhere reported the matching of need to services [29].

We have demonstrated that it is possible to deliver this level of quality of service consistently over a sustained period of six years.

### Limitations

We cannot identify the extent to which routine psychiatric in-reach services based on a referral system identified all those with severe mental illnesses prior to the introduction of this two stage screening and court liaison system. We can however compare the numbers ascertained using this system with the numbers found using a research-standard methodology in the same prison two years prior to the commencement of this service.

This study was confined to one prison, though the prison concerned accounted for 58.5% of all remand committals nationally over the six year period studied, including Dublin, the largest city by far in Ireland. The denominators for the numbers detected and diverted are derived from Irish Prison Service statistics and in some respects may be of varying quality year on year. The number diverted in 2011 underestimates the total number diverted from those who screened positive in 2011, as some of those were not diverted until early in 2012.

We have not reported on the nature of the offences committed in this paper, though we have reported this elsewhere [29]. We intend to report on longer term outcomes including re-committal of those diverted from prison to mental health services in a separate paper.

We have not reported the time taken to achieve diversions here. We will report this in a subsequent analysis of factors influencing collaboration between forensic and community services, as well as addressing other research gaps identified on systematic review of jail diversion programs in other jurisdictions [30].

### Interpretation

This observational study has shown that consistent quality can be maintained over time in this model of service delivery. Because this is an action research study, one of the advantages of the process has been a notably positive attitude towards the ascertainment of mental illness amongst remand committals and the diversion of such persons to mental health services. This represents a form of cultural change in the prison.

It is interesting to note also that the proportion of men committed to prison on remand who were found to be psychotic has varied very little over the six year period of observation. The period of observation commenced some years after the major decarceration of patients from old-style asylums to community care, though numbers of available psychiatric beds continue to fall [31].

### Generalisability

The success of this service has drawn favorable comment from the Inspector of Prisons in annual reports [32]. This model of service depends on providing staffing of sufficient experience in sufficient numbers to be available at least five days a week. A sufficient volume of remand committals is necessary to make this service both clinically effective in terms of numbers diverted, and cost effective. There are three small prisons distant from the capital that provide for remanded and sentenced prisoners. It would probably not be viable to provide a team-based service such as this for such relatively small numbers.

The service does however provide an invaluable training resource for specialist forensic practitioners.

## Conclusions

A landmark paper on Mental Health in prisons internationally by Fazel and Baillargeon [5] recommended that greater health-care resources should be targeted at prisons since they provide “a rare public health opportunity” for screening and treatment [5]. While ideally, diversion services should be delivered at the earliest stage of contact with the criminal justice system, such as police stations [14,33] and courts [1-3,34,35], the centralized model described here provides for a standardized and equitable approach for large population aggregates, as well as economies of scale through integration with prison inreach services for remand prisoners.

## Competing interests

The authors declare that they have no competing interests.

## Authors’ contributions

All authors were involved in the action research process. CO’N wrote the first draft of the manuscript. HGK advised on study design and statistical analysis. All authors read and approved the final manuscript.
